# Understanding the Functional Properties of Neonatal Dendritic Cells: A Doorway to Enhance Vaccine Effectiveness?

**DOI:** 10.3389/fimmu.2018.03123

**Published:** 2019-01-10

**Authors:** Nikos E. Papaioannou, Maria Pasztoi, Barbara U. Schraml

**Affiliations:** ^1^Biomedical Center, Institute for Cardiovascular Physiology and Pathophysiology, Ludwig-Maximilians-University Munich, Munich, Germany; ^2^Walter-Brendel-Centre of Experimental Medicine, University Hospital, Ludwig-Maximilians-University Munich, Munich, Germany

**Keywords:** dendritic cell (DC), DC subsets, early life immunity, vaccination, immune system development, T cell activation, innate immunity, DC targeting

## Abstract

Increased susceptibility to infectious diseases is a hallmark of the neonatal period of life that is generally attributed to a relative immaturity of the immune system. Dendritic cells (DCs) are innate immune sentinels with vital roles in the initiation and orchestration of immune responses, thus, constituting a promising target for promoting neonatal immunity. However, as is the case for other immune cells, neonatal DCs have been suggested to be functionally immature compared to their adult counterparts. Here we review some of the unique aspects of neonatal DCs that shape immune responses in early life and speculate whether the functional properties of neonatal DCs could be exploited or manipulated to promote more effective vaccination in early life.

## Introduction

Early life immune balance is essential for survival and establishment of healthy immunity in later life. The neonatal period in mammals represents a critical window, in which the immune system has to keep a fine balance between efficient pathogen defense and maintenance of tolerance against a continuous flood of commensal microbes and environmental antigens (1–4). Reduced inflammatory capacity is an inherent feature of early life immunity that has been attributed to an altered repertoire of immune cells, as well as a relative functional immaturity of immune cells in early compared to later life (2–5). For example, the T and B cell pools are not fully expanded at birth and are biased to generate T helper (Th) 2 type responses compared to a more Th1 type response in adults (1). Neonatal, but not adult, monocytes and neutrophils potently suppress T cell activation *in vitro* and therefore strongly resemble myeloid derived suppressor cells (6). Although neutrophil-like myeloid derived suppressor cells show microbicidal activity (6), the inflammation-induced trafficking of neutrophils, as well as their ability to form extracellular traps, are reduced in fetal and early life compared to adult (7–9). Fetal monocytes are transcriptionally distinct from their adult counterparts and fetal, as well as, neonatal monocytes show distinct responsiveness to inflammatory stimuli than adult monocytes (10–14). Macrophages first develop before birth and are thought to aid in tissue remodeling during development, whereas they acquire their full-blown immune functions with increasing age (11, 14). Microglia of the brain for instance gain an immune-related gene signature over time and in response to microbial signals (15). Additionally, the existence of specific immune regulatory cells of erythroid origin in early life has been suggested to dampen inflammatory responses (4, 16).

As a result of these immune alterations, neonates exhibit an increased susceptibility to infections (2, 5). In humans, pathogens that are often asymptomatic in adults, such as *Haemophilus influenzae* type B, *Bordetella pertussis* and *Streptococcus pneumoniae*, account for the death of more than two million infants per year world-wide (17). Dendritic cells (DCs) have been implicated to promote immune responses to these pathogens in adults (18–21). It is possible to immunize infants under one year against these pathogens, but a single immunization does not necessarily provide immediate protection, or as in the case of *H. influenzae* type B, antibody titers may not persist (17). For other pathogens, such as rotavirus, immunization is first possible few weeks after birth leaving infants at risk of infection, when the disease is most severe (17, 22). The efficacy, success and challenges of vaccines in early life, as well as existing efforts to improve their effectiveness have recently been reviewed elsewhere (17). Here we focus on the functional differences between DCs in early and adult life. DCs sense the presence of pathogens or damage via so called pattern recognition receptors (PRRs) and initiate innate, as well as adaptive immune responses through cytokine production and antigen presentation (23–25). In their function as immune sentinels DCs have been extensively targeted to increase vaccine effectiveness and DC based vaccines hold promise in adults (26). However, as other immune cells, DCs in early life differ from their adult counterparts in phenotype and function, raising the question, whether targeting DCs could be used to elicit protective immunity in infants and increase vaccine effectiveness. Although most of the data discussed here derive from mouse studies, parallels likely exist in humans, as DC subsets and functions appear highly conserved across species (23, 25).

## Dendritic Cells Develop as Functionally Distinct Subsets

Among DCs, we distinguish two main functionally and developmentally distinct cell lineages. Conventional or classical DCs (cDCs) are remarkable activators of adaptive immune responses with a remarkable capacity to capture, process, and present antigens to T cells (23–25, 27). Plasmacytoid DCs (pDCs) on the other hand are critical for defense against viruses, because of their capacity to respond to viral antigens and secrete type I interferons (IFN) (28, 29). Most of our knowledge about the development of these cells is based on studies in adult mice. In adults, DCs have a short lifespan and rely on constant replenishment from bone marrow-derived hematopoietic stem cells (30, 31). cDCs and pDCs were long thought to derive from a common myeloid precursor, the so-called common dendritic cell progenitor (CDP) (32, 33). Within this progenitor fraction, expression of the C type lectin receptor CLEC9A/DNGR-1 distinguishes cells with cDC-restricted developmental potential (34). These cDC restricted CDPs further differentiate into pre-cDCs, which leave the bone marrow and seed lymphoid and non-lymphoid tissues (31, 35) where they differentiate into the two main cDC1 and cDC2 subsets in response to environmental cues (23–25, 27). Of note, the signals that regulate cDC differentiation in tissues remain poorly defined and recent studies indicate that the commitment of pre-cDCs toward cDC1 or cDC2 may already be imprinted in the bone marrow (36, 37). In contrast to cDCs, pDCs exit the bone marrow as fully differentiated cells (38) and only a fraction of pDCs appears to belong to the myeloid lineage, whereas the majority of pDCs arises from lymphoid progenitors (39).

In adults cDC1 and cDC2 are developmentally and functionally distinct cell subsets that can be distinguished based on their differential dependence on transcription factors (23–25, 27). While cDC1 rely on BATF3, ID2 and IRF8 for their development, cDC2 require the transcription factors IRF4, ZEB2, and RELB and are additionally influenced by Notch2 signaling and retinoic acid (23–25, 27). Although not yet investigated in detail, at least some of these developmental pathways are conserved with age, as cDC1-like cells are missing in spleen, mesenteric lymph node and intestinal lamina propria of neonatal BATF3-deficient mice (40, 41). Additionally, DCs in early and late life require FMS-like tyrosine kinase 3 ligand (FLT3L) for their development (42, 43). In adults cDC1 can be reliably identified across tissues by expression of XCR-1, DNGR-1, and CD205 (23–25, 27). In addition, CD8α and CD24 mark cDC1 in lymphoid tissues (23–25, 27). The integrin CD103 marks cDC1 in non-lymphoid tissues, although it is also expressed on a subset of intestinal cDC2 (23–25, 27). cDC2 on the other hand are marked by expression of CD11b, CD172a, and CLEC4A4 (23–25, 27). Functionally, cDC1 are exceptional activators of CD8^+^ T cells, in part for their superior activity to cross-present cell associated antigens (23–25, 27). cDC1 are additionally dominant inducers of Th1 polarized immune responses due to their strong capacity to produce IL-12 (23–25, 27). In contrast, cDC2 are generally thought to be more efficient at activating CD4^+^ T cell and inducing Th2- or Th17-biased effector responses (23, 24, 44, 45). Since cDC1 and cDC2 have unique functions in immunity and can be distinguished by expression of select surface markers, they are attractive targets for the manipulation of immune responses in adults (26).

## Altered Subset Distribution of Dendritic Cells in Early Life

Although DCs reportedly can be found in mice as early as embryonic day 17, the DC compartment of newborn mice is not fully developed and subject to dynamic age-dependent changes during development (46, 47). In mice the neonatal period includes the first 10 days after birth, which correlates to the first 28 days of life in humans (1, 17). However, it is important to note that in terms of immune development there is substantial temporal variation between mice and humans in early life (48). In murine neonates the DC compartment in lymphoid and non-lymphoid organs is much smaller than that of adults and hallmarked by distinct DC subset distribution (46, 47). The frequency of splenic cDCs at birth is about ten-fold lower than that of adult spleen and, similarly, neonatal splenic pDCs are seven-fold lower in terms of frequency compared to their adult counterparts (46, 47). This is also reflected in lower numbers of DCs in neonatal spleen, which is not fully developed at birth in terms of organ architecture and size (46, 47). By about 5 weeks of age, when the total splenic cellularity reaches adult levels, both cDC and pDC numbers also reach adult levels (46, 47). A relative scarcity of DCs in newborn mice is also evident in other organs, such as thymus, lymph nodes, lung and intestine (47, 49–53). Altered immune responses and infection susceptibility in early life could therefore simply be a by-product of low DC numbers. Administration of the DC growth factor FLT3L leads to a strong increase in DC numbers in neonatal mice and results in increased resistance to *Listeria monocytogenes* (*L. monocytogenes)* and herpes simplex virus 1 (HSV-1) (43). Similarly, administration of FLT3L significantly enhances resistance of neonates to the intestinal parasite *Cryptosporidium parvum* by increasing the number of intestinal CD103^+^ cDCs, which include both cDC1 and a fraction of cDC2 (54).

**Figure 1:**
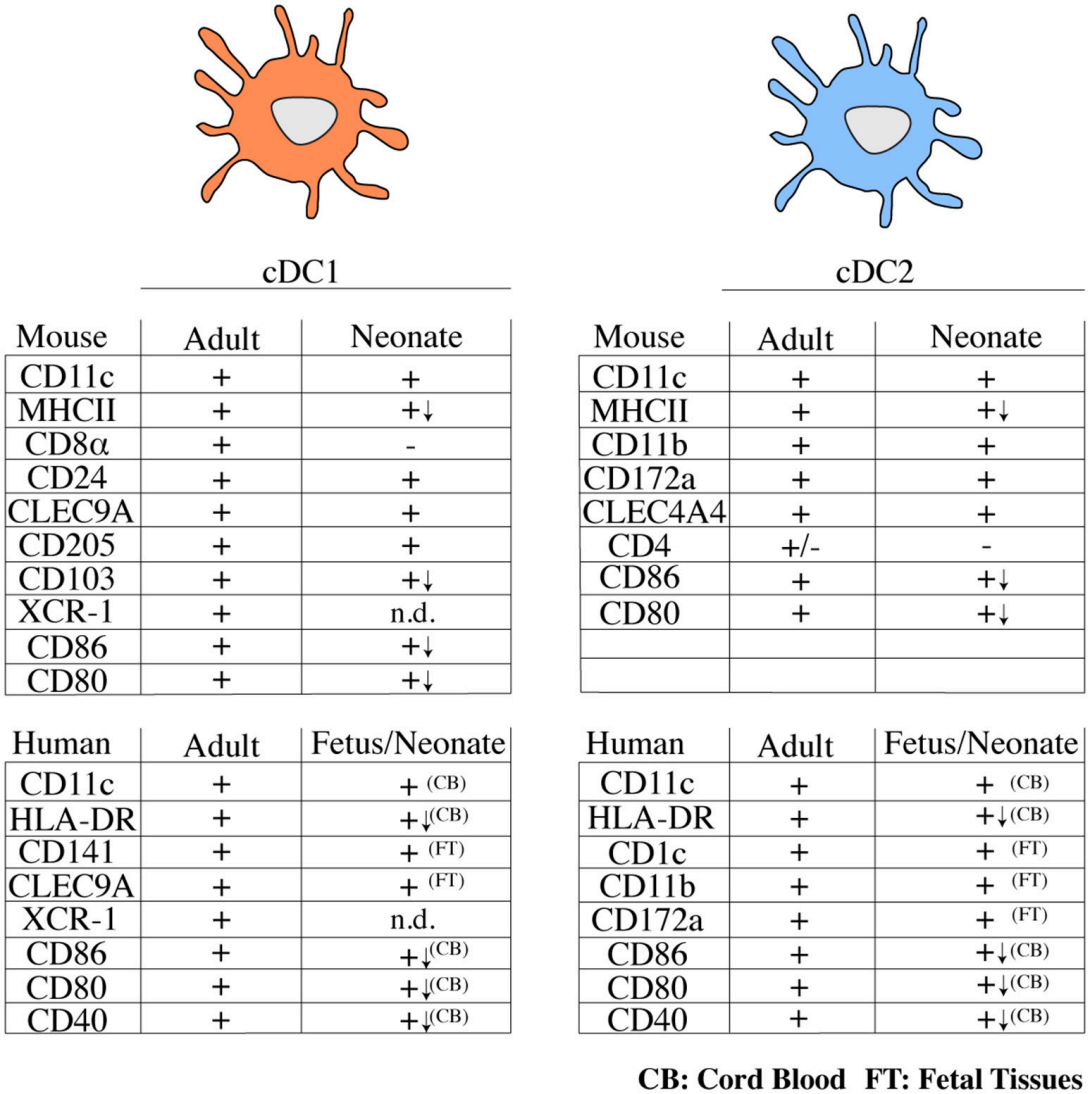
Overview of typical surface markers expressed on cDC subsets from mice and humans in early and adult life. (+) marker is expressed, (–) marker is not expressed or is expressed by a small fraction of cells, n.d., not determined; (↓) lower expression; CB, cord blood; FT, fetal tissues; cDC, conventional dendritic cell; MHCII, major histocompatibility complex class II; CLEC, C-type lectin domain family; HLA, human leukocyte antigen.

In adults, the cDC compartment is dominated by cDC2, while in early life cDC1 appear to be the dominant cDC subtype in spleen and lymph nodes (46, 47, 51). In thymus, cDC1 remain the dominant cDC population also in adults, possibly owing to a unique requirement of this cell type in ensuring central T cell tolerance (55). A systematic analysis of DC subset distribution with age across non-lymphoid organs has not been performed, with one exception being the lung, where cDC1 outnumber cDC2 in neonates, but this relationship is inversed in adults (50). It is important to note that most of these studies relied on the use of surface markers to identify cDC subsets. Notably, within the first 6 days of life, cDC1 from spleen and mesenteric lymph node lack CD8α, although they do express CD24, CLEC9A/DNGR-1, and CD205 (40, 46, 47). Expression of XCR-1 on neonatal cDC1 has not been investigated. These data have led to the suggestion that CD8α^−^ cDC1 may represent a progenitor of bona fide cDC1 (40) and indicate that the use of surface markers to define cDC subsets in early life needs to be approached with caution. A summary of surface markers expressed on neonatal and adult DCs can be found in Figure 1.

Why DC subset distribution differs between neonates and adults is unclear. It is possible that DC differentiation may be intrinsically programmed to generate a functionally adapted DC repertoire that meets the needs of immune responses in early life. However, age-specific changes in specific organ environments could alter DC subset development. cDC1 in neonatal mediastinal lymph nodes express lower levels of CD103 than cDC1 from adult mediastinal lymph nodes (49). This difference in CD103 expression has been attributed to the unique cytokine environment of the lung in early life, such as low expression of GM-CSF (49). Thus, the DC compartment of neonates differs from that of adults in terms of cell number, subset distribution and marker expression.

## Dendritic Cell Function in Neonates vs. Adults

It is well established that neonatal DCs in both mice and men are functionally distinct from DCs in adults, which has been suggested to represent a level of functional immaturity. In mice, early life cDCs produce lower levels of pro-inflammatory cytokines than their adult counterparts. In the first week of life, splenic cDC1 produce lower amounts of IL-12 in response to CpG or after *in vivo* poly I:C treatment (40, 47). Similarly, splenic cDC2 produce less IFN-γ after stimulation with IL-12 and IL-18 compared to their adult counterparts and pDCs produce less IFN-α after combined treatment with CpG, GM-CSF, IL-4 and IFN-γ than pDCs from 6-week old mice (47). Interestingly, when cDC1 from Balb/C rather than C57BL/6 mice were analyzed, CpG-induced IL-12 production did not differ between cDC1 from 7-day old and adult mice (46). In cDC1 from C57BL/6 mice CpG-induced IL-12 production in early life can be augmented by addition of GM-CSF, IFN-γ, and IL-4 to culture conditions, however, the level of IL-12 produced still does not reach that of adult cDC1 (40, 47). These data indicate that some pathogen sensing pathways are fully functional in early life cDCs and that cytokine production of neonatal cDCs may at least in part be augmented through the use of additional costimulatory signals, which in turn could be used to boost immune responses in early life.

A key property of DCs is their ability to activate naïve T cells. Early life cDCs express lower basal levels of major histocompatibility complex class II (MHCII) and costimulatory molecules compared to adult cDCs (43, 47, 49) but expression of these molecules can be induced, for instance upon CpG stimulation *in vivo* (46). Despite these differences, splenic cDC1 and cDC2 from 1-week-old mice are able to induce allogeneic CD4^+^ T cell proliferation *in vitro* to a similar extent as the same subsets from 6-week old mice (47). In the first week of life, CD8α^−^ cDC1 phagocytose *L. monocytogenes* and cross-present *L. monocytogenes*-derived antigens to CD8^+^ T cells as efficiently as adult cDC1 (40). However, while cDC1 from adults respond to this pathogen in a predominantly pro-inflammatory manner, neonatal cDC1 additionally produce the anti-inflammatory cytokine IL-10, which suppresses CD8^+^ T cell activation (40). Accordingly, IL-10 blockade augments the antigen-specific CD8^+^ T cell proliferation induced by neonatal CD8α^−^ cDC1 *in vitro* (40). Whether this IL-10 production has an impact on pathogen burden *in vivo* has not been examined, but these results indicate that the response to *L. monocytogenes* is intrinsically different between cDC1 from neonatal and adult mice. Following infection with respiratory syncytial virus (RSV) 7-day-old mice show an altered CD8^+^ T cell response to that is hallmarked by an epitope shift toward D^b^M_187−195_, rather than the K^d^M2_82−90_ epitope that is immunodominant in adults (49). This epitope shift can be partially rescued by administration of costimulatory signals *in vivo* (51), suggesting that lower levels of costimulatory molecules on cDCs contribute to the observed epitope bias. However, it is noteworthy, that cDC1 from 7-day-old RSV infected mice preferentially present D^b^M_187−195_ epitopes (49, 51), indicating that early life cDCs may exhibit intrinsic differences in antigen processing. Notably, epitope bias may also be found in humans, as infants infected with RSV show age-dependent differences in antibody specificities (56). cDC1 are also required for generating antigen-specific CD8^+^ T cell responses to rotavirus, a major cause of childhood gastroenteritis (41). Interestingly, neonatal Batf3-deficient mice have a stronger impairment in the antigen-specific CD8^+^ T cell response to rotavirus than adults, yet, Batf3 deficiency delays viral clearance only minimally in neonates (41). These data suggest, that *in vivo* other immune mechanisms are put in place that compensate for a lack of cDC1 in this case.

Antigen exposure in early life can elicit both Th1 and Th2 responses (1, 57, 58), however generates a strong bias for Th2 recall responses later in life. This has been partially attributed to a T cell intrinsic Th2 bias in early life but also to the altered cytokine production of cDCs in the first weeks of life, such as low-level IL-12 production (59, 60). Immunization with OVA before postnatal day 6 leads to an upregulation of IL-13 receptor α (IL-13Rα) in antigen specific Th1 cells (59). During secondary exposure to OVA, IL-13Rα expression renders antigen-specific Th1 cells sensitive to IL-4-induced apoptosis, thus leading to Th2-biased recall responses (58, 59). Exogenous administration of IL-12 or adoptive transfer of IL-12 competent cDCs from adult mice reverses the Th2-biased recall response (59), indicating that the low level IL-12 production by cDCs in early life exerts lasting effects on immunity. Why functional differences between neonatal and adult DCs exist, is unclear but several studies suggest that the neonatal environment functionally imprints DCs. As an example, in the developing lung IL-33 produced by epithelial cells during alveolarization on postnatal day 14 suppresses the ability of pulmonary cDC2 to produce the Th1 cytokine IL-12 (50). IL-33 instead promotes OX40L expression, which in turn leads to a stronger ability of cDC2 to promote Th2-biased responses and allergy (50). Th17 responses can be mounted in neonatal mice, for instance after infection with *Yersinia enterolytica* (61), but early life Th17 responses are damped by T cell derived IL-4 (62). The role of neonatal DCs in this process has not been investigated in detail.

Functional differences between early life and adult DCs have also been observed in humans. cDC2 from fetal spleen respond differently to various PAMPs than adult splenic cDC2 and as in mice, the response in the fetus is marked by higher production of anti-inflammatory cytokines (63). Although human fetal splenic cDC2s can induce allogeneic T cell proliferation, they limit T-cell-derived TNF-α production in an arginase 2-dependent manner and promote differentiation of Foxp3^+^ regulatory T cells (63). pDCs from cord blood exhibit a severe defect in IFN-I secretion upon TLR9 stimulation with CpG when compared to pDCs from peripheral blood in adults, whereas the cytokine response to influenza A or human immunodeficiency virus is similar (64, 65). Collectively, these data show that the response of neonatal DCs to pathogens differs from that of adults in many ways, however, some signaling pathways induce immune responses that are comparable to those in adults.

## Neonatal Dendritic Cells as Targets for Vaccination?

**Figure 2:**
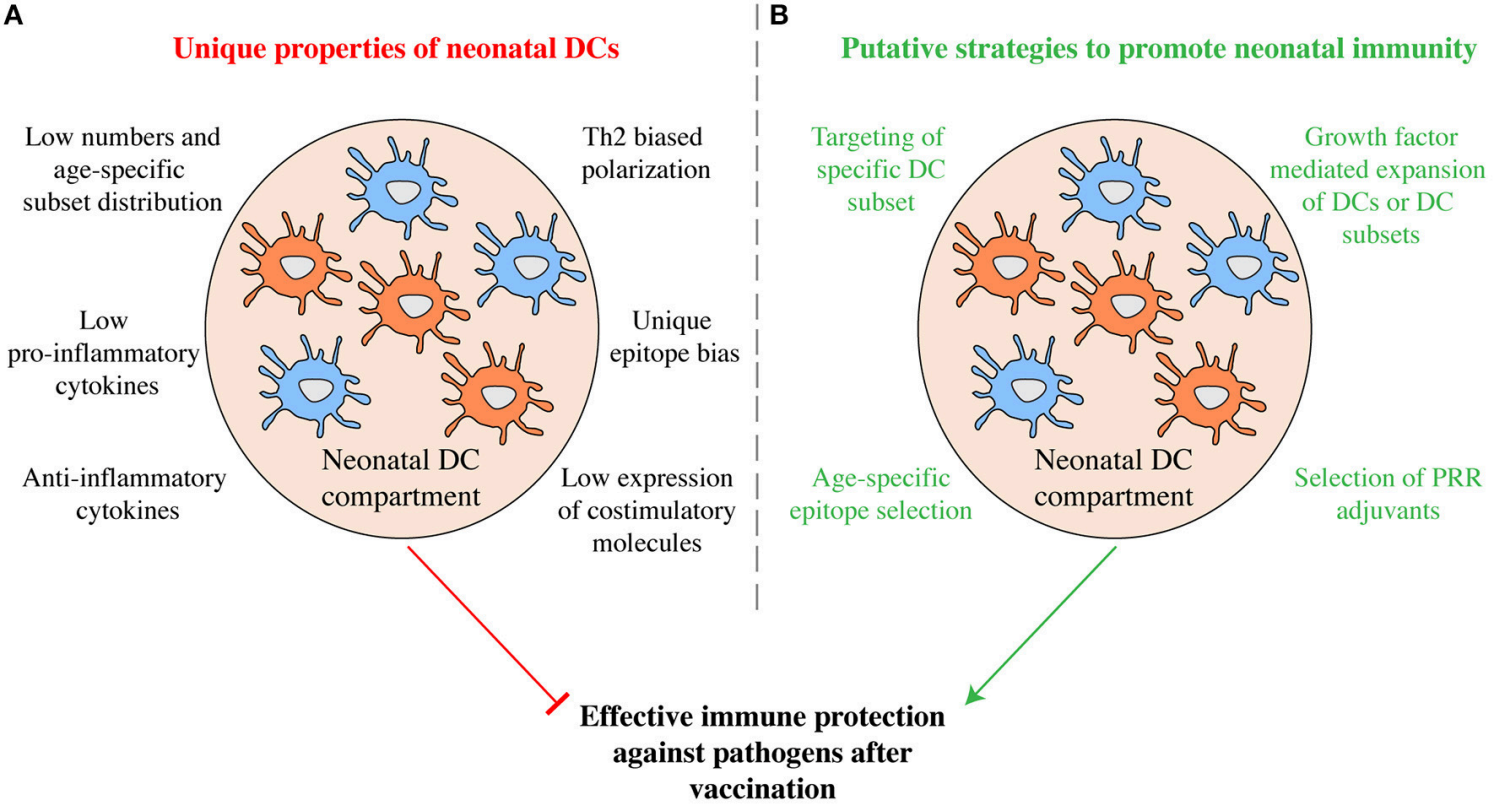
Manipulation of the neonatal DC compartment as putative strategy to promote immunity. **(A)** Some of the functional properties that characterize neonatal DCs are shown. Taken together they may inhibit efficient immunity in currently used vaccination protocols. **(B)** Exploiting the unique functions of neonatal DCs may prove promising in developing more efficient vaccination protocols tailored to early life. Through age-specific epitope selection, DC subset specific epitope delivery, the combinatorial use of select PRR agonists as adjuvants, as well as via the manipulation of the DC compartment using growth factors, increased protective humoral and cellular immunity may be promoted.

The unique properties of neonatal cDCs discussed above likely contribute to the relative ineffectiveness of vaccines in early life (Figure 2A) and it will be important to determine to what extent tailoring vaccines to the properties of early life DCs can be used to boost immunity. Similarities in surface receptor expression exist between neonatal and adult cDC subsets in both mice and humans (Figure 1) and specific targeting of cDC subsets holds promise for immunization in adults (26). Subset specific targeting using selectively expressed surface receptors can induce protective immunity in murine neonates. Targeting OVA to cDC1 via antibodies directed against CLEC9A with poly I:C as adjuvant on postnatal day 3 efficiently protects murine neonates against lethal challenge with OVA-expressing *L. monocytogenes* in adulthood (40). Whether targeting cDC2 in early life can induce protective immunity remains to be investigated. The prominent capacity of cDC2 to migrate to draining lymph nodes from the lung (50) indicates that they may be potent targets for initiating T cell responses. But several functions of neonatal cDC2 have not been studied in detail and it is unclear whether they induce effector T cell responses and promote T follicular helper cell differentiation and concomitant antibody production as efficiently as their adult counterparts.

Synergistic use of TLR agonists greatly increases the Th1 polarizing capacity of human adult DCs (66) and thus the use of defined PRR agonists may be used to promote immunity in early life. A recent study showed that stimulation with multiple TLR agonists elicits stronger secretion of Th1 polarizing cytokines from total human cord blood mononuclear cells than stimulation with a single TLR agonist, however, in cord blood cDCs a single TLR agonist induced stronger pro-inflammatory cytokine production than a combinatorial treatment (67). Agonists of the stimulator of interferon genes (STING) induce expression of costimulatory molecules on neonatal bone marrow derived DCs *in vitro* and promote secretion of IFN-β (68). *In vivo* administration of STING agonists in alum in neonatal mice promotes germinal center formation, IFN-γ production by antigen specific T cells, as well as increased antibody titers (68). Taken together these data highlight that the correct choice of adjuvant, either alone or in combination, is important for the design of effective vaccine strategies (17).

Immunization critically depends on the pathogen epitope selected to be vaccinated against. In neonatal mice, T cell responses are directed against a distinct set of antigenic epitopes compared to adults (49, 51). Similarly, in human infants neutralizing antibodies against RSV are preferentially generated against a distinct array of viral epitopes than in adults (56). Thus, selection of age-specific epitopes may also serve as a strategy to foster early life immunity and may help to circumvent deprivation of antigen by pre-existing maternal antibodies (17). At the same time, expanding the DC compartment or specific DC subsets using growth factors may ultimately shift epitope bias and cytokine production. Understanding the functional and developmental properties of neonatal cDCs and how to manipulate them may potentially be used to increase the effectiveness of neonatal immunization beyond what is possible today (Figure 2B). However, immune responses in early life are complex and vaccine effectiveness is influenced by a wide array of factors, including preexisting maternal antibodies (17). Therefore, further studies, especially in humans, are required to better understand the unique aspects of DC development and function in neonates and in early life, as well as the interplay of DCs with other components of the immune system, in order to fully capture whether DCs could be exploited to alter early life vaccination.

## Author Contributions

NP and BS wrote the manuscript with assistance from MP. NP designed the figures. All authors provided critical feedback throughout the writing process.

### Conflict of Interest Statement

The authors declare that the research was conducted in the absence of any commercial or financial relationships that could be construed as a potential conflict of interest.
